# Translatome analysis of Tuberous Sclerosis Complex-1 patient-derived neural progenitor cells reveal rapamycin-dependent and independent alterations

**DOI:** 10.21203/rs.3.rs-2702044/v1

**Published:** 2023-03-27

**Authors:** Inci S. Aksoylu, Pauline Martin, Francis Robert, Krzysztof J. Szkop, Nicholas E. Redmond, Shan Chen, Roberta L. Beauchamp, Irene Nobeli, Jerry Pelletier, Ola Larsson, Vijaya Ramesh

**Affiliations:** 1 Department of Oncology-Pathology, Science for Life Laboratory, Karolinska Institute, Stockholm, Sweden;; 2 Ctr. for Genomic Med., Department of Neurology, Massachusetts Gen. Hosp., Boston, MA;; 3 Department of Biochem. and Goodman Cancer Res. Ctr., McGill Univ., Montreal, QC, Canada;; 4 Department of Biol. Sciences, Inst. of Structural and Mol. Biology, Birkbeck, Univ. of London, London, United Kingdom;; 5 These authors contributed equally to this work.

## Abstract

Tuberous sclerosis complex (TSC) is an inherited neurocutaneous disorder caused by mutations in *TSC1* or *TSC2* genes, with patients often exhibiting neurodevelopmental (ND) manifestations termed TSC-associated neuropsychiatric disorders (TAND) including autism spectrum disorder (ASD). The hamartin-tuberin (TSC1-TSC2) protein complex inactivates mechanistic target of rapamycin complex 1 (mTORC1) signaling, leading to increased protein synthesis via inactivation of translational repressor eIF4E-binding proteins (4E-BPs). In *TSC1*-null neural progenitor cells (NPCs), we previously reported early ND phenotypic changes, including increased proliferation/altered neurite outgrowth, which were unaffected by mTORC1-inhibitor rapamycin. Here, using polysome-profiling to quantify translational efficiencies at a transcriptome-wide level, we observed numerous TSC1-dependent alterations in NPCs, largely recapitulated in post-mortem brains from ASD donors. Although rapamycin partially reversed TSC1-associated alterations, most neural activity/synaptic- or ASD-related genes remained insensitive but were inhibited by third-generation bi-steric, mTORC1-selective inhibitor RMC-6272, which also reversed altered ND phenotypes. Together these data reveal potential implications for treatment of TAND.

## INTRODUCTION

Tuberous sclerosis complex (TSC) is an inherited multisystem disorder involving a range of symptoms including epilepsy, autism spectrum disorder (ASD), intellectual disability (ID), and slow growing hamartomas in many organs. TSC is caused by mutations in the *TSC1* or *TSC2* genes, encoding the tumor suppressor proteins hamartin (TSC1) and tuberin (TSC2)^1,2^. The TSC proteins act as a central hub relaying signals from diverse cellular pathways to control mammalian/mechanistic target of rapamycin complex 1 (mTORC1) activity, which regulates cell growth and proliferation^3,4^. Aberrant activation of mTORC1 in TSC has led to rapamycin (Rap) analogs (“rapalogs”) emerging as a lifelong therapy for TSC hamartomas, as their discontinuation leads to resumed growth of TSC-associated lesions^5–8^. Recent clinical trials revealed that rapalogs reduce epilepsy in 40% of TSC patients^9^. In contrast, rapalogs are ineffective in treating TSC-associated neuropsychiatric defects (TAND) and autism^10,11^. Therefore, new treatments for TSC that are superior to rapalogs with respect to anti-proliferative effects in tumors, and efficacy toward the non-tumor CNS manifestations of the disorder are needed.

Several mouse models of TSC have provided valuable clues regarding neurological symptoms, but incompletely recapitulate the human phenotypes^12^. Recent studies examining the role of TSC1 or TSC2 have employed genetically engineered human embryonic stem cell lines with heterozygous or homozygous loss of *TSC2*; TSC patient-derived induced pluripotent stem cells (iPSCs); or isogenic gene-edited iPSCs from patients with *TSC1* or *TSC2* mutations that have been differentiated into e.g. neural progenitor cells (NPCs), forebrain neurons, cerebellar Purkinje neurons, astrocytes or oligodendrocytes^13–21^. Many phenotypic alterations, including somatic hypertrophy, increased dendritic arborization, augmented proliferation rate, altered electrophysiology and hyper-activation of mTORC1, are more pronounced in *TSC1*-null or *TSC2-*null cells when compared with heterozygous or wild-type (WT) counterparts (reviewed in^22,23^). Furthermore, transcriptome analyses have revealed ample alterations when comparing isogenic gene-edited *TSC1-* or *TSC2*-null NPCs or neurons to heterozygous or WT cells^14,15,17,19^.

Among its many activities^24^, mTORC1 plays a major role in regulating gene expression by modulating how efficiently mRNAs are translated into proteins. Consistent with a key role of mRNA translation in determining proteome composition, translatomes (commonly defined as the pool of mRNA associated with ribosomes)^25^ resemble proteomes more closely than corresponding transcriptomes^26–28^. mTORC1 regulates cap-dependent translation by modulating the assembly of eukaryotic initiation factor (eIF) 4F, a complex consisting of a cap binding protein (eIF4E), a DEAD-box RNA helicase (eIF4A), and a large scaffolding protein (eIF4G). mTORC1 activation leads to direct phosphorylation of two key substrates involved in regulating translation initiation: eIF4E binding proteins (4E-BPs) and ribosomal protein p70S6 kinases (S6Ks). 4E-BPs are a family of translation inhibitors consisting of three members, the best studied being 4E-BP1, which when de-phosphorylated competes with eIF4G for binding to eIF4E and prevents eIF4F complex formation. Once phosphorylated, 4E-BPs dissociate from eIF4E, facilitating eIF4F complex formation^29^. Further, activation of S6K by mTORC1 also affects translation initiation by: (i) increasing eIF4A availability through phosphorylation and degradation of a negative repressor, PDCD4, and (ii) phosphorylating eIF4B which stimulates eIF4A helicase activity and promotes initiation complex formation^30,31^. Interestingly, a recent study reported that mTORC1-driven translation is high in human pluripotent stem cells and is suppressed during neural differentiation^32^. Moreover, numerous changes in mRNA translation without corresponding changes in mRNA levels have been observed across human neuronal development, highlighting the importance of translational control for developing neurons^32^.

Our comparisons of transcriptomes between isogenic NPCs revealed a quantitative genotype-dependent response whereby genes upregulated/downregulated in *TSC1*-heterozygous NPCs were further increased/decreased in *TSC1*-null cells when compared to genetically matched CRISPR-corrected WT cells. Interestingly, this included genes linked to ASD, epilepsy and ID^17^. However, despite alterations in mRNA translation being a major mechanism modulating gene expression downstream of mTOR in cancer cells^33^, translatome studies are lacking in TSC stem cell models. Recent studies have documented that early neurodevelopmental events, such as NPC proliferation, neurite outgrowth and migration, that precede synaptogenesis also play a role in disease pathogenesis of ASD and other neuropsychiatric disorders^34–40^. The enhanced proliferation and neurite outgrowth consistently observed in *TSC1*-null NPCs when compared with isogenic WT controls suggest that this may underlie early neurodevelopmental defects in TSC^17^.

Using our isogenic NPC model generated from TSC1 patient-derived iPSCs, we identified TSC1-sensitive mRNA expression and translation. Strikingly, TSC1-sensitive mRNA translation observed in NPCs was recapitulated in human ASD brain samples from the Brodmann area 19 when contrasted to controls. Furthermore, although polysome-profiling revealed a partial reversal of TSC1-sensitive translation upon rapamycin treatment, most genes related to neural activity/synaptic regulation or ASD showed rapamycin-insensitive translation. However, these genes could be reversed by the third-generation bi-steric mTORC1-selective inhibitor RMC-6272, which more efficiently suppresses 4E-BP1-phosphorylation in NPCs when compared to rapamycin^41^. This was accompanied by reversal of rapamycin-insensitive phenotypes in *TSC1*-null NPCs, suggesting that more efficient targeting of mTORC1 may be an attractive treatment strategy in ASD.

## RESULTS

### *TSC1* loss leads to widespread alterations of mRNA translation in patient-derived isogenic neural progenitor cells (NPCs)

**Figure 1.**
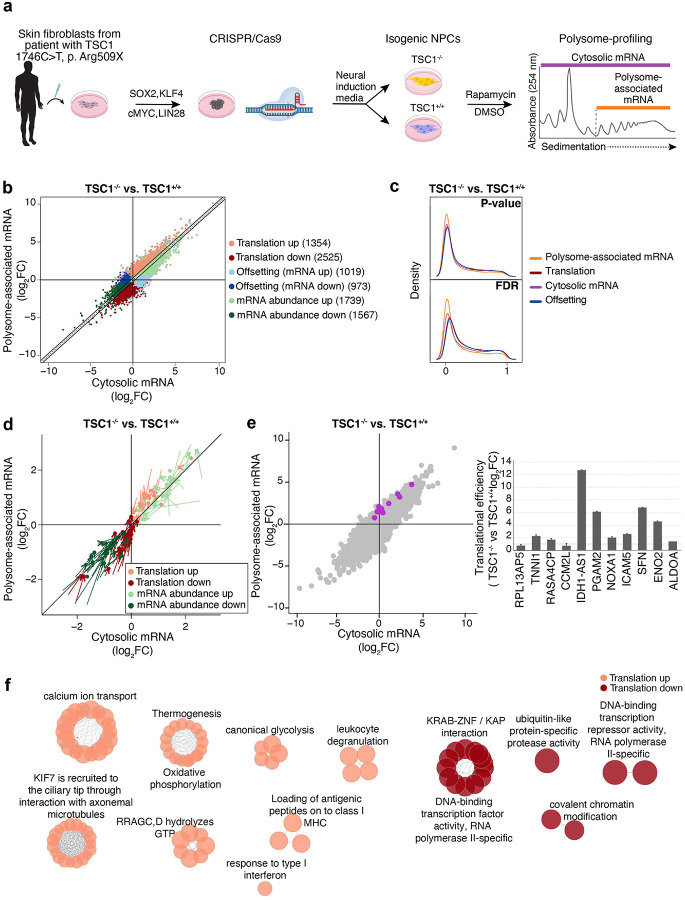
TSC1-associated alterations in mRNA abundance and translation. **a.** Overview of the polysome-profiling approach in NPCs to identify TSC1-associated changes in gene expression. **b.** Scatter plot from anota2seq analysis comparing polysome-associated to cytosolic mRNA log_2_ fold-changes for the *TSC1*^*−/−*^ NPC vs *TSC1*^+/+^ NPC comparison (n=3). Genes identified as differentially regulated by anota2seq (see Methods for applied thresholds) in each gene expression mode are visualized in the scatter, and the total number of genes is indicated in brackets. **c.** Kernel densities of p-values or FDRs (from anota2seq analysis) for the comparison of *TSC1*^−/−^ vs *TSC1*^+/+^ NPCs. Densities are shown for analysis of polysome-associated RNA, cytosolic RNA, translation and offsetting. **d.** Scatter plot of log2 fold-changes comparing *TSC1*^−/−^ vs *TSC1*^+/+^ NPCs estimated using NanoString nCounter assays (n=4). Each gene is represented by an arrow where the start of the arrow shows the fold-changes estimated by RNA sequencing (i.e. Fig. 1B) and the end of the arrow indicates the fold-change obtained by NanoString nCounter assays. **e**. Scatter plot from anota2seq analysis comparing polysome-associated to cytosolic mRNA log_2_ fold-changes for the *TSC1*^*−/−*^ NPC vs *TSC1*^+/+^ NPC comparison (left panel) showing genes randomly selected for validation by RT-qPCR (purple) relative to background (grey) from 3 biological replicates. The right panel indicates changes in translational efficiencies (i.e. polysome-associated mRNA normalized to cytosolic mRNA) according to RT-qPCR for the same genes. **f.** Gene-set enrichment analysis for proteins encoded by mRNAs whose translation increase or decrease in *TSC1*^−/−^ vs *TSC1*^+/+^ NPCs. Each node corresponds to a process or pathway and edges connect nodes that were identified from overlapping genes.

To determine the impact of *TSC1* loss-of-function mutations on mRNA levels and translation in NPCs, we used skin fibroblasts from a patient with a heterozygous mutation in *TSC1* exon 15 (1746C>T, Arg509X) to derive isogenic *TSC1*-null NPCs (−/−) and corrected *TSC1*-WT NPCs (+/+) as described previously^17^ (Fig. 1a, Fig. S3a). This isogenic cell pair was then used to assess TSC1-associated changes in gene expression at multiple levels using polysome-profiling^42^. During polysome-profiling, total cytosolic mRNA is fractionated depending on ribosome association and a pool of polysome-associated mRNA is isolated in parallel with total cytosolic mRNA (Fig. 1a). The resulting RNA pools from *TSC1*^−/−^ and *TSC1*^+/+^ NPCs were quantified using RNA sequencing followed by analysis using anota2seq^43,44^ to identify three modes of TSC1-associated gene expression alterations: (i) changes in polysome-associated mRNA not paralleled by corresponding alterations in total mRNA levels (denoted “translation” and, under conditions when translation elongation is unaffected, interpreted as changes in translational efficiency leading to modulation of protein levels); (ii) congruent changes of polysome-associated and total mRNA (denoted “abundance” representing alterations in mRNA levels impacting protein levels downstream of e.g. modulation of transcription and/or mRNA-stability); and (iii) alterations in total mRNA not paralleled by corresponding changes in polysome-associated mRNA (denoted “offsetting“ and interpreted as instances where mRNA translation opposes alterations in protein levels imposed by modulation of mRNA levels [discussed in detail elsewhere]^45,46^). As visualized by a scatterplot comparing TSC1-associated changes in total cytosolic and polysome-associated mRNA (Fig. 1b), and densities of p-values or FDRs (Fig. 1c) from anota2seq analysis, this revealed numerous TSC1-associated alterations in translation and abundance together with instances of translational offsetting. To validate the observed changes in gene expression, we selected 200 genes whose translation or abundance was increased or decreased in *TSC1*^−/−^ as compared to *TSC1*^+/+^ NPCs and quantified their expression pattern using the NanoString nCounter Gene Expression Analysis technology. This largely confirmed the gene expression modes (Fig. 1d). To further validate changes in mRNA translation, we focused on a subset of genes that did or did not show accompanying alterations in mRNA levels (Fig. 1e, left), and performed RT-qPCR using total or polysome-associated mRNA as input. This identified larger TSC1-associated changes in polysome-associated mRNA as compared to total mRNA for all genes in the validation subset (Fig. 1e, right). Next, we assessed the potential functional impact of these changes in translational efficiencies using ClueGO-based gene ontology analysis^47^. This revealed that translation of mRNAs encoding proteins annotated to e.g. ion transport, immune system function, RNA polymerase II and oxidative phosphorylation were selectively altered (Fig. 1f; Table S1a-b). Overall, these data show that loss of TSC1 function in NPCs leads to reprogrammed gene expression via alterations in both mRNA abundance and translation.

### Alterations of mRNA translation in ASD patient compared with control brains

**Figure 2.**
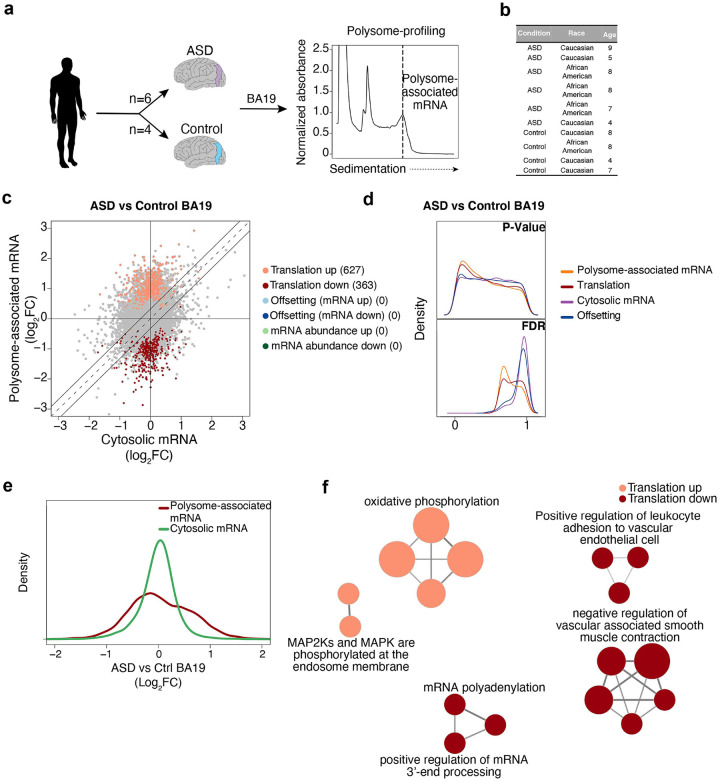
ASD-associated alterations of mRNA translation in BA19. **a.** Overview of experimental setup for polysome-profiling of BA19 brain samples. Polysome-tracings were obtained using the optimized sucrose gradient (as described in Methods). **b.** Characteristics of ASD-affected donors and controls included in the study. **c.** Scatter plot comparing polysome-associated to cytosolic mRNA log_2_ fold-changes for the ASD vs Control BA19 (similar to Fig. 1b). **d.** Kernel densities of p-values or FDRs (from anota2seq analysis) for the comparison of ASD vs Control BA19 (similar to Fig. 1c). **e.** Kernel densities of log_2_ fold-changes for total cytosolic and polysome-associated mRNA comparing ASD vs Control BA19. **f.** Gene ontology analysis (similar to Fig. 1f) for the comparison of ASD to Control BA19.

We next sought to assess whether similar patterns of mRNA translation to those identified as TSC1-associated in NPCs are observed in post-mortem brain tissue from ASD patients. To this end, we obtained 10 brain samples collected from neurotypical and ASD-affected donors matched for age (4–9 years old) and gender (male). Furthermore, all samples originated from the Brodmann Area 19 (BA19), a part of the occipital lobe cortex involved in responses to visual stimuli (Fig. 2a–b). As brain samples were small, we used a recently developed optimized polysome-profiling technique amenable to small tissue samples^48^ to obtain, to our knowledge, a first dataset of transcriptome-wide alterations of mRNA translation in ASD-affected post-mortem brain tissue. Similarly to above (Fig. 1b–c), we analyzed the resulting dataset using anota2seq^43,44^ (Fig. 2c–d). Despite the limited statistical power to detect gene expression changes due to the scarce availability of matched ASD and control samples, we observed an enrichment of low p-values for ASD-associated alterations in translation (Fig. 2d). This was further supported by a left-shifted distribution of FDRs (Fig. 2d) together with a larger range of fold changes (ASD vs control) for polysome-associated mRNA as compared to total cytosolic mRNA (Fig. 2e). Strikingly, ClueGO gene ontology analysis revealed that genes regulated via translation are involved in previously ASD-implicated functions including oxidative phosphorylation, MAPK pathway and alternative polyadenylation (Fig. 2f; Table S1c-d). Therefore, albeit limited by the low availability of tissues for studies, these data support reprogrammed translation in post-mortem brain tissue from donors affected by ASD.

### *TSC1*^−/−^ NPCs recapitulate mRNA translation in brains of ASD patients

**Figure 3.**
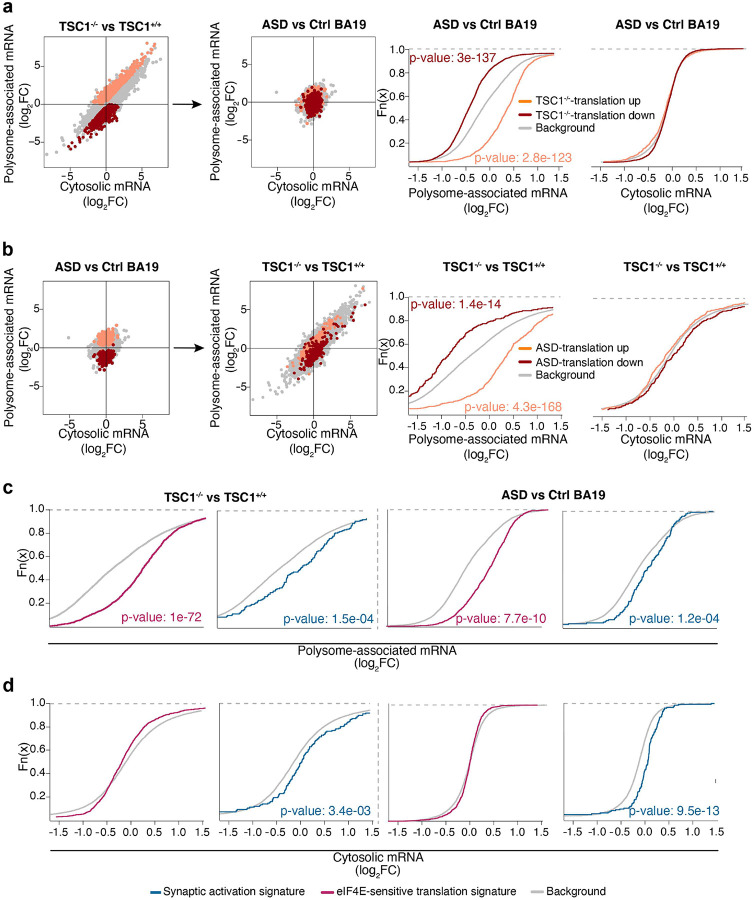
*TSC1*^−/−^ NPCs recapitulate translation observed in ASD BA19 samples. **a.** Scatter plots from anota2seq analysis (left two panels) where transcripts whose translation was activated or suppressed in *TSC1*^−/−^ vs *TSC1*^+/+^ NPCs (i.e. from Fig. 1b) are indicated in *TSC1*^−/−^ vs *TSC1*^+/+^ NPCs and ASD vs control comparisons. Shown are also empirical cumulative distribution function (ECDF) plots assessing regulation of the same gene sets relative to the background (i.e. genes not in gene set) for polysome-associated and cytosolic mRNA log_2_ fold-changes (ASD vs control BA19; two rightmost plots). Wilcoxon rank-sum test p-vales are indicated for the comparison of each gene set relative to the background. **b.** Scatter plots and ECDF plots (similar to Fig. 3a) assessing regulation of transcripts whose translation were altered in ASD vs control BA19 in the comparison of *TSC1*^−/−^ vs *TSC1*^+/+^ NPCs. **c-d.** ECDF plots assessing genes related to synaptic activation and transcripts whose translation increased upon eIF4E overexpression. Signatures were evaluated in *TSC1*^−/−^ vs *TSC1*^+/+^ NPCs (left two panels) and ASD vs control BA19 (right two panels). Fold-changes were calculated using polysome-associated mRNA (**c**) or cytosolic mRNA (**d**). Wilcoxon rank-sum test p-vales are indicated for the comparison of each gene set relative to the background (i.e. genes not in gene sets).

We compared the data sets obtained from NPCs and BA19 samples to determine if there are overlaps in gene expression programs. First, we assessed whether transcripts showing TSC1-sensitive translation in NPCs showed altered gene expression when comparing samples originating from ASD vs control brains. Strikingly, transcripts whose translation was increased when comparing *TSC1*^−/−^ to *TSC1*^+/+^ NPCs (i.e. those identified in Fig. 1b) showed increased translation also when comparing ASD to controls (as levels of polysome-associated mRNA were increased while total mRNA levels where unchanged; Fig. 3a). Similarly, transcripts that were translationally suppressed in *TSC1*^−/−^ relative to *TSC1*^+/+^ NPCs were also translationally suppressed in BA19 from ASD relative to control subjects (Fig. 3a). We next performed the reciprocal analysis by assessing whether transcripts with altered translation in ASD vs control samples (i.e. those identified in Fig. 3a) also showed TSC1-sensitive expression in NPCs. Indeed, transcripts showing increased or decreased translation when comparing BA19 samples from ASD to controls revealed similar translation patterns when comparing *TSC1*^−/−^ to *TSC1*^+/+^ NPCs (Fig. 3b). Accordingly, the NPC model captures alterations in mRNA translation occurring in brains of ASD patients.

To further explore similarities between the two data sets, we assessed whether synaptic genes (Table S2a)^49^, and transcripts whose translation was previously identified as induced upon overexpression of eIF4E (”eIF4E-sensitive”)^50^, are regulated in the NPC and ASD datasets. In agreement with hyperactivation of the mTORC1/eIF4E axis, previously identified eIF4E-sensitive transcripts were translationally activated in *TSC1*^−/−^ relative to *TSC1*^+/+^ NPCs as well as in ASD vs control brains (as judged by their increased polysome-association in the absence of changes in total mRNA; Fig. 3c–d; Table S2b). In contrast, synaptic genes showed increased levels of both polysome-associated and cytosolic mRNA in both datasets (Fig. 3c–d). These findings further underline that a more complete understanding of ASD-associated gene expression changes and their mechanistic underpinnings requires studies of mRNA translation.

### Rapamycin only partially reverses TSC1-associated translation

**Figure 4.**
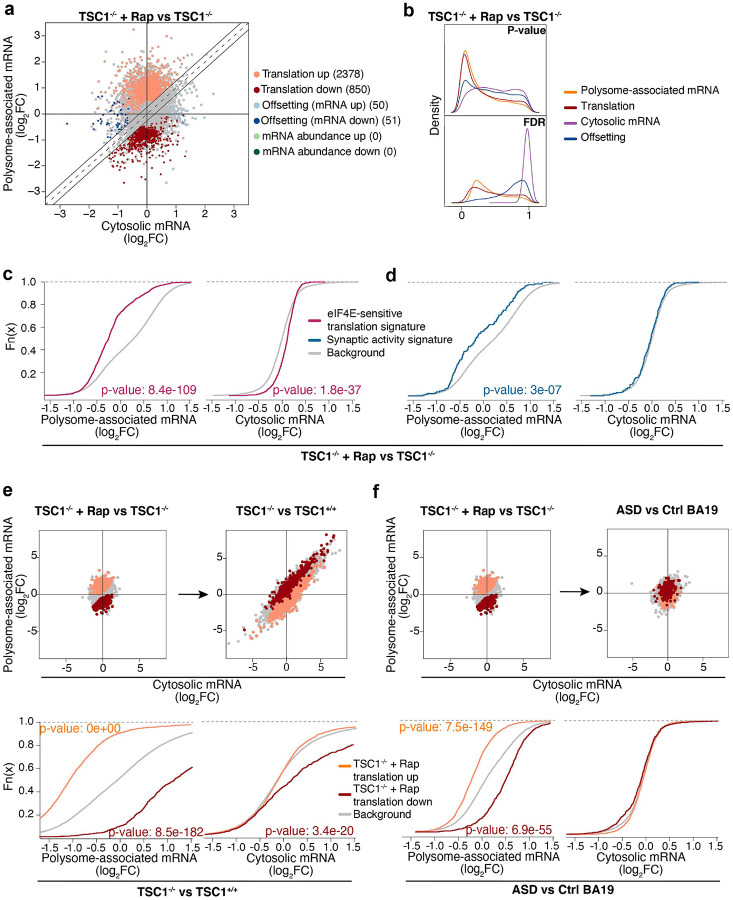
Rapamycin partially reverses TSC1- and ASD-associated translation. **a-b.** Comparison of *TSC1*^−/−^ cells in presence vs absence of rapamycin (similar to Fig. 1B–C). **c-d.** ECDF plots for rapamycin sensitivity of eIF4E- and synaptic activity-signatures in *TSC1*^−/−^ cells (similar to Fig. 3c–d). **e-f.** Scatter plots (similar to Fig. 3a) assessing how transcripts showing rapamycin-sensitive translation are modulated in the *TSC1*^−/−^ vs *TSC1*^+/+^ NPCs (**e**) or ASD vs control BA19 (**f**) comparisons.

As discussed above, TSC1 loss leads to hyperactivated mTORC1 signaling, which affects mRNA translation both globally and selectively^33,51^. Accordingly, mTORC1 inhibitors have been considered as a strategy to treat phenotypes resulting from loss of TSC1^5,6,8^. To assess whether these agents reverse TSC1-associated mRNA translation in NPCs, we used polysome-profiling in cells treated with the mTORC1 inhibitor rapamycin (Fig. 1a). Anota2seq analysis comparing *TSC1*^−/−^ NPCs in the presence or absence of rapamycin revealed that short-term treatment almost exclusively modulated mRNA translation (Fig. 4a–b). Consistent with rapamycin inhibiting translation via the mTORC1/eIF4E axis, translation of mRNAs previously identified as eIF4E-sensitive (same subset as in Fig. 3c–d) was suppressed in rapamycin-treated *TSC1*^−/−^ NPCs (Fig. 4c, Table S2b). In addition, rapamycin reduced polysome-association of mRNAs transcribed from some genes with synaptic activity (same subset as in Fig. 3c–d) (Fig. 4d, Table S2a). Next, using the same strategy as above (Fig. 3a–b), we assessed whether identified rapamycin-sensitive translation is modulated when comparing *TSC1*^−/−^ vs *TSC1*^+/+^ NPCs or ASD vs control brains. Consistent with hyperactivation of eIF4E-sensitive translation downstream of mTORC1 (Fig. 3c–d), transcripts with suppressed translation upon rapamycin treatment showed hyperactive translation when contrasting *TSC1*^−/−^ to *TSC1*^+/+^ NPCs or ASD vs control brains (Fig. 4e–f).

**Figure 5.**
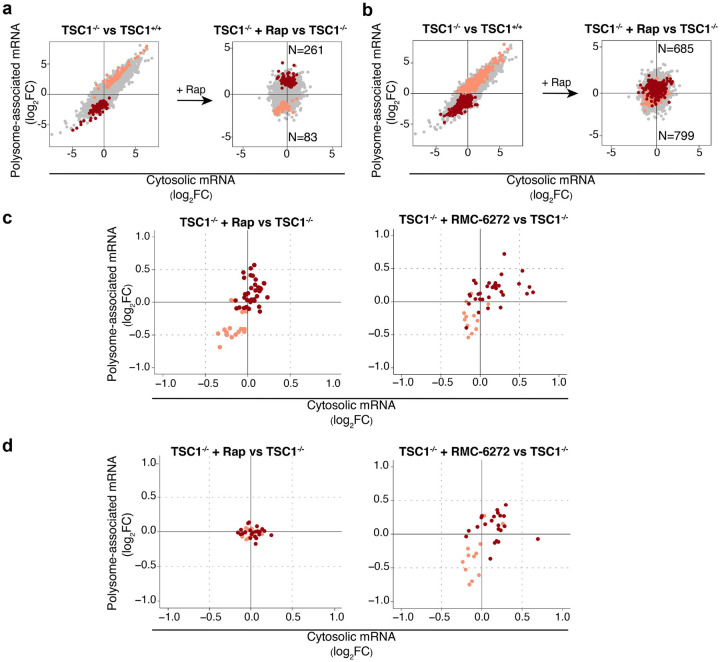
RMC-6272 reverses rapamycin-insensitive TSC1-associated alterations in mRNA translation. **a-b.** Scatter plots (similar to Fig. 3a) of transcripts showing TSC1-associated mRNA translation that were reversed (**a**) or insensitive (**b**) to rapamycin treatment. The number of transcripts following each pattern of regulation is indicated. **c-d.** Scatter plot of log2 fold-changes from NanoString nCounter assays for transcripts showing TSC1-associated mRNA translation that was sensitive (**c, left**) or insensitive (**d, left**) to rapamycin (according to RNA sequencing-based quantification) but sensitive to RMC-6272 (c and d, right). Fold-changes were calculated between cells treated with rapamycin (left) or RMC-6272 (right) relative to control (DMSO).

Although rapamycin reverses TSC1-associated changes of mRNA translation in NPCs (Fig. 4e), previous studies have indicated that the effect on the translatome is only partial - likely due to rapamycin incompletely reducing phosphorylation of 4E-BPs^52^. Consistent with incomplete reversal of mTORC1-sensitive translation by rapamycin, neurite outgrowth in *TSC1*^−/−^ cells was not rescued by rapamycin^17^. To assess the extent to which TSC1-associated translation is sensitive to rapamycin, we separated mRNAs whose translation was altered in *TSC1*^−/−^ vs *TSC1*^+/+^ NPCs into subsets with rapamycin-reversed (Fig. 5a) or -insensitive translation (Fig. 5b). Indeed, this revealed that only a subset of TSC1-associated translation changes was reversed by rapamycin, while most transcripts were insensitive. A new generation of bi-steric mTORC1-selective inhibitors, which suppresses phosphorylation of 4E-BP1 to a greater extent, has recently been developed^41,53^. To evaluate whether these inhibitors may reverse TSC1-associated mRNA translation more efficiently than rapamycin, we used our NanoString nCounter Gene Expression Analysis code set to analyze effects on translation in *TSC1*^−/−^ NPCs treated with RMC-6272, an mTORC1-selective bi-steric third-generation inhibitor^41,54^ compared to rapamycin. We focused the analysis on NanoString targets with TSC1-associated changes in mRNA translation, separated these into those whose translation was reversed or insensitive to rapamycin, and compared the effects of mTOR allosteric inhibition with mTORC1 bi-steric inhibition. As expected, transcripts showing rapamycin-sensitive translation were also sensitive to RMC-6272 (Fig. 5c). Conversely, transcripts whose translation was insensitive to rapamycin (Fig. 5d, left) were largely sensitive to RMC-6272 (Fig. 5d, right). Accordingly, these studies suggest that more efficient inhibition of mTORC1 with a bi-steric inhibitor reverses TSC1-associated alterations in mRNA translation to a greater extent than rapamycin and may therefore have distinct effects on ASD-associated phenotypes.

### TSC1 NPCs show genotype-dependent phenotypes that are reversed by RMC-6272

**Figure 6.**
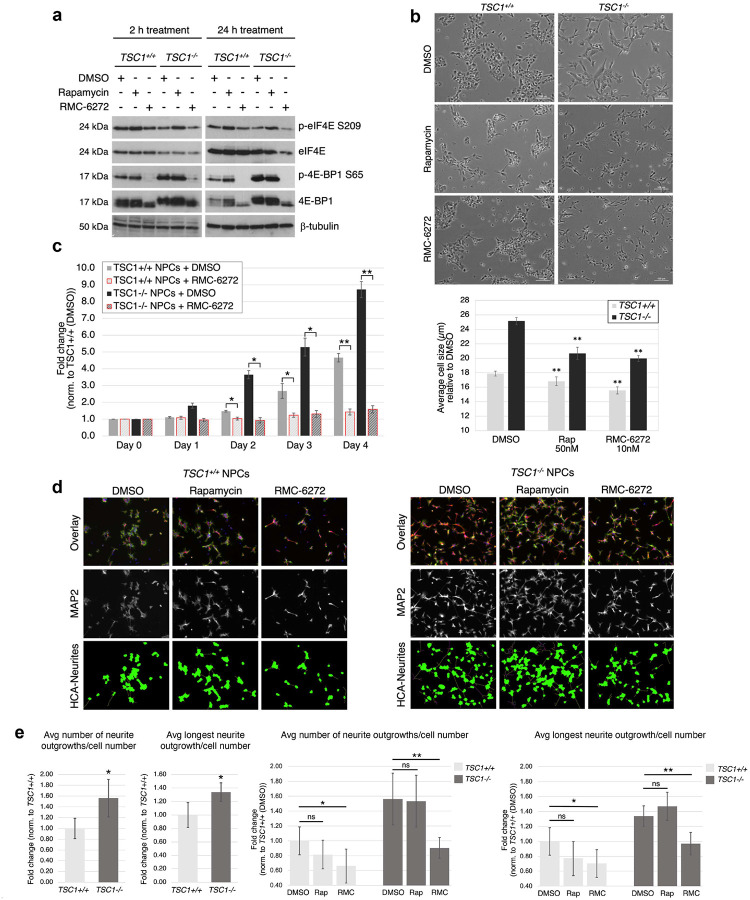
Treatment of TSC1 NPCs with RMC-6272 shows superior inhibition of early neurodevelopmental phenotypes as compared with rapamycin. **a.** Immunoblotting for indicated proteins in *TSC1*^+/+^ and *TSC1*^*−/−*^ NPCs treated with 10 nM RMC-6272 or 50 nM rapamycin (2 or 24h, n=3). Tubulin serves as a loading control. **b.** Bright field images and quantification of *TSC1*^+/+^ and *TSC1*^*−/−*^ NPCs treated with 50 nM rapamycin or 10 nM RMC-6272 to assess cell size (n=3) is shown. **c.** Proliferation rate for NPCs after treatment with DMSO or 10 nM RMC-6272 was quantified for live cells by trypan blue-exclusion at Day 0 (D0, cell number at seeding), and live cell numbers were assessed at D1–4. Data represents average fold change normalized to *TSC1*^+/+^ NPCs treated with DMSO at D0 (n=3 ± SD). **d-e**. MAP2 immunofluorescence staining (**d**) shows increased neurite outgrowth (number and length) in *TSC1*^*−/−*^ NPCs (right panel) vs *TSC1*^+/+^ NPCs (left panel) along with effects on neurite outgrowth upon treatment with 50 nM rapamycin or 10 nM RMC-6272 (RMC). Quantitation using a custom image analysis pipeline and HCA Vision imaging software creating neurite segmentation is shown (**d** and **e**) from 4 field images/treatment (~50 cells/field). Data normalized to DMSO-treated *TSC1*^+/+^ NPCs (mean ± SEM). *p<0.05, **p<0.001, ns = not significant, calculated by Student’s t test.

We previously reported increased cell size as well as early neurodevelopmental phenotypes, including proliferation rate and neurite outgrowth, in *TSC1*^−/−^ as compared with *TSC1*^+/+^ NPCs. Rapamycin treatment, while decreasing cell size, did not rescue the increased proliferation or neurite outgrowth^17^. Based on our results indicating that a subset of rapamycin-insensitive genes with genotype-dependent altered translation were reversed by RMC-6272 (Fig. 5d), we tested the ability of RMC-6272 to rescue early neurodevelopmental phenotypes in *TSC1*^−/−^ NPCs. Immunoblotting of *TSC1*^+/+^ and *TSC1*^−/−^ NPCs treated with rapamycin or RMC-6272 for 2 or 24 h showed attenuation of phosphorylated ribosomal S6 (p-S6 S240/244) but only RMC-6272 reversed phosphorylation of 4E-BP1. In addition, consistent with our previous report^17^, p-eIF4E was increased upon rapamycin treatment, but remained unchanged in RMC-6272 treated cells (Fig. 6a, Fig. S3b). Furthermore, treatment of NPCs with 50 nM rapamycin or 10 nM RMC-6272 for 24 h led to a similar reduction in cell size when compared with DMSO-treated control cells (Fig. 6b). As reported previously^17^, proliferation and neurite outgrowth were unaffected by rapamycin treatment. In contrast, RMC-6272 rescued both these phenotypes. A 4-day treatment with 10 nM RMC-6272 inhibited proliferation of *TSC1*^+/+^ and *TSC1*^−/−^ NPCs, as determined by viable cell counts using trypan blue exclusion, with cell numbers at days 1–4 (D1–4) remaining close to seeding (D0; Fig. 6c). Furthermore, as we have previously shown, *TSC1*^−/−^ NPCs displayed a significant increase in both neurite number and length compared to *TSC1*^+/+^ NPCs (Fig. 6d–e, left panel). Strikingly, treatment with RMC-6272 led to a significant reduction in neurite number and length, compared with DMSO-treated cells, while rapamycin did not affect the number or length of neurites (Fig. 6d–e, right panel). Taken together, these data support that treatment with the third-generation mTORC1 inhibitor leads to more complete inhibition of mTORC1 downstream targets including 4E-BP1 and is more potent than rapamycin in rescuing the altered early neurodevelopmental phenotypes such as proliferation and neurite outgrowth in *TSC1*-mutant NPCs.

## DISCUSSION

It is well established that loss of TSC1 or TSC2 results in activation of mTORC1 signaling, which has led to FDA approval for treatment of TSC-associated tumors with first-generation mTORC1 inhibitors such as rapalogs everolimus/RAD-001. However, rapalogs have not been very effective for treating TSC-associated neuropsychiatric defects and autism^10,11^. The mTORC1 signaling pathway plays a critical role in protein synthesis in normal cells including stem cells, and in human disease through regulation of translation initiation (reviewed in^55,56^).The mTORC1/eIF4F axis is therefore critical in shaping the proteome. Although transcriptome-wide studies of TSC-associated mRNA translation have been performed in mouse embryonic fibroblasts^57^, the effects of TSC-loss in patient-derived NPCs have not been assessed. Here we sought to bridge this gap in knowledge.

Here we, for the first time, reveal the complex pattern of gene expression alterations downstream of TSC1 loss in patient-derived NPCs encompassing both changes in mRNA abundance as well as numerous alterations in translational efficiencies. Interestingly, TSC1-dependent alterations in mRNA translation observed in NPCs were largely recapitulated in human ASD brains. In addition, our study of TSC1-associated gene expression also indicated ample translational offsetting, which denotes a poorly characterized gene expression mode possibly representing adaptation^45,46^. Although this may be of interest to fully understand how TSC1 loss reprograms gene expression, as this mode of regulation was not observed in human ASD brains, we did not study it further herein. Furthermore, although polysome-profiling revealed a partial reversal of TSC1-associated gene expression alterations following rapamycin treatment, most genes related to neural activity/synaptic regulation or ASD that showed TSC1-dependent translation were rapamycin-insensitive. Among mTOR inhibitors, first-generation allosteric rapalogs effectively suppress phosphorylation of mTORC1 target S6K1, but not 4E-BP1 in many cell types. Furthermore, allosteric rapalogs activate AKT, a downstream target of mTORC2, by negative feedback loops^24^, which prompted development of second-generation, orthosteric mTOR kinase inhibitors (active site mTOR inhibitors) including Torin 1, AZD8055 and TAK-228/MLN0128, which potently inhibit both mTORC1 and mTORC2. As mTORC2 promotes lipogenesis, glucose uptake and cell survival through downstream targets AKT and SGK, active site mTOR inhibitors appears to be more toxic than rapalogs^58^. Orthosteric mTOR inhibitors efficiently reduce phosphorylation of 4E-BP1 when compared with rapalogs, but exhibit short residence time compared to rapamycin, resulting in poor *in vivo* efficacy^33^. The limited clinical benefits of first- and second-generation mTOR inhibitors led to the recent development of a third-generation mTORC1-directed inhibitor RapaLink-1. As a prototype of the bi-steric class of mTOR inhibitors, RapaLink-1 links the high affinity of rapamycin for mTORC1 with the effective active site mTOR inhibition of TAK-228^33^. RapaLink-1 was shown to be highly potent in reducing phosphorylation of both S6K1 and 4E-BP1 while retaining approximately 4-fold selectivity for mTORC1 as compared to mTORC2. RapaLink-1 also showed antitumor efficacy in glioma models *in vivo* with no significant toxicities^33,58^. More recent bi-steric compounds show higher selectivity for mTORC1 over mTORC2 (more than 30-fold selectivity), along with potent suppression of 4E-BP1 phosphorylation^41,53^. These bi-steric mTORC1-selective inhibitors, including RMC-6272 and its clinical counterpart RMC-5552, show strong antitumor activity either alone or when combined with other treatments in several preclinical cancer models. RMC-5552 also demonstrates preliminary evidence of anti-tumor activity at tolerated doses^41,54^. Here we reveal that RMC-6272 is not only more potent than rapamycin in inhibiting mTORC1, but also reverses some of the translational changes not reversed by rapamycin (Fig. 5d). These findings are consistent with previous comparisons between the effects of rapamycin and the active site mTOR inhibitor PP242 on transcriptome-wide translation in cancer cells^52^. More importantly, unlike rapamycin, RMC-6272 can rescue early neurodevelopmental phenotypes such as proliferation and neurite outgrowth in *TSC1*^*−/−*^ NPCs (Fig. 6 and^17^), raising the question whether 4E-BP1-dependent translation could be essential for some of the neurodevelopmental phenotypes in TSC and other mTORC1-activated neurodevelopmental disorders.

In addition to TSC, dysregulated mTORC1 signaling is also observed in other syndromic ASDs such as PTEN hamartoma syndrome, Fragile X syndrome, RASopathies including NF1, Angelman syndrome and Rett syndrome, as well as idiopathic ASD^59,60^, raising the possibility that cap-dependent translation downstream of mTORC1 could play an essential role in neurodevelopmental and neuropsychiatric disorders. Many of the recent large-scale studies have focused on the transcriptome for understanding gene expression changes in the pathophysiology of ASD and other neuropsychiatric disorders^61–63^. Defining changes in mRNA translation in neurodevelopmental and neuropsychiatric disorders remains largely unexplored and our study here describing such changes in TSC1 patient-derived neural progenitor cells will likely open avenues for correlating transcriptional alterations with changes in mRNA translation in ASD and other neurodevelopmental disorders with dysregulated mTORC1 signaling.

## METHODS

### Cell lines and reagents

TSC1 patient-derived NPCs, including *TSC1*-null (−/−) and CRISPR-corrected *TSC1*-WT (+/+), along with culture conditions, have been previously described^17^. Rapamycin was from EMD Millipore (Burlington, MA), and RMC-6272 (previously known as RM-006) was generously provided by Revolution Medicines, Inc. (Redwood City, CA). All antibodies are listed in Table S3.

### Polysome fractionation and RT-qPCR for NPCs

Lysate preparation for polysome-profiling from 4 biological replicates was carried out as previously described^64^. Briefly, TSC1 NPC lines were seeded at 40,000 cells/cm^2^, with a total of 5×10^7^ cells seeded per drug treatment condition. The next day after seeding, cells were treated for 2h with 50 nM rapamycin, 10 nM RMC-6272, or DMSO as a vehicle control. Treated cells were then rinsed with 1X PBS containing 100μg/ml cycloheximide (CHX) (Sigma, St. Louis, MO), harvested by scraping on ice in PBS/CHX, and pelleted by centrifugation at 300g for 10min at +4C. Cell pellets were lysed in 10 mM Tris-HCl (pH 8), 140 mM NaCl, 1.5 mM MgCl_2_, 0.1% sodium deoxycholate, 0.1% Triton X-100, 1 mM DTT and 100 μg/ml CHX. Lysates were cleared by spinning for 2 min at 13000g and quickly frozen on dry ice. When ready for processing, lysates were thawed and loaded onto a 10–50% sucrose gradient, centrifuged for 2h15 min at 35,000 rpm in a SW41 rotor using a Sorvall Discovery 90SE centrifuge. The gradients were fractionated on a Teledyne ISCO Foxy R1 apparatus while monitoring the OD_254_. Fractions corresponding to mRNA associated with more than two ribosomes were pooled and the RNA extracted using TRIzol (Thermo Fisher, Waltham, MA) according to the manufacturer’s protocol. Prior to loading samples on the sucrose gradient, RNA was extracted from 10% of the lysate using TRIzol, and the resulting RNA was denoted as total RNA. RNA sequencing libraries were prepared from the resulting samples using Illumina v2.5 Kits and sequenced (3 biological replicates) on an Illumina NextSeq 500 at the Canada’s Michael Smith Genome Sciences Centre (BC Cancer Research Institute, Vancouver, Canada).

### Polysome fractionation of post-mortem brain samples

Polysome fractionation of post-mortem Brodmann area 19 samples from ASD-affected donors (n=6) and matched controls (n=4) provided by NIH NeuroBiobank was performed using an optimized sucrose gradient, as previously described^42,48^. RNA sequencing libraries were generated using the Smart-seq2 protocol as described previously^48^. Single-end 51 base sequencing was performed using the HiSeq2500 platform and the HiSeq Rapid SBS kit v2 chemistry at the National Genomics Infrastructure, Science for Life Laboratory, Stockholm, Sweden. Bcl to fastq conversion was performed using bclfastq_v2.19.1.403 from the CASAVA suite.

### Analysis using anota2seq

Genes with 0 mapped RNA sequencing read in one or more samples were discarded resulting in analysis of 12950 and 11998 genes in post-mortem brain samples and NPCs, respectively. The data was TMM-log2 normalized and analyzed using anota2seq^44^ (v. 1.14.0; parameters: minSlopeTranslation = −1, minSlopeBuffering = −2, maxSlopeTranslation = 2, maxSlopeBuffering = 1, deltaPT = deltaTP = deltaP = deltaT = log2(1.2))^43,44^. During analysis of the NPC dataset, replicate was included in the model to correct for batch effects and three contrasts were assessed, namely (i) *TSC1*^*−/−*^ vs *TSC1*^+/+^ , (ii) *TSC1*^*−/−*^ + rapamycin vs *TSC1*^*−/−*^ and (iii) *TSC1*^+/+^ + rapamycin vs *TSC1*^+/+^, with the following thresholds to identify differentially expressed genes: minEff = log2(1.5) and maxRvmPadj = 0.15 [i.e. fold change>log2(1.5) and FDR<0.15]. In the post-mortem dataset, 6 ASD-affected brains were compared to 4 neurotypical, and relaxed thresholds were applied: minEff=log2(1.25) and pVal = 0.05.

### RNA-Seq data preprocessing and quality control

Quality of sequencing reads (paired-end for NPC data set; single-end for post-mortem brain data set) was confirmed using FastQC (v0.11.4), available online at: http://www.bioinformatics.babraham.ac.uk/projects/fastqc/; BBmap (v. 36.59) https://www.osti.gov/servlets/purl/1241166 (parameters: k = 13, ktrim=n, useshortkmers=t, mink = 5, qtrim=t, trimq = 10, minlength = 25) was used to trim reads for Illumina Truseq and Nextera adapter sequences and low-quality base calls. For the NPC data set, BBmap (v. 36.59) was used to remove sequencing reads mapping to rRNA sequences obtained from the SILVA ribosomal RNA gene database^65^. The resulting reads were aligned to the human reference genome (build hg38) using HISAT2 (v.2.1.0 and v.2.0.4 for NPC and post-mortem brain datasets, respectively, using, in addition to default parameters, “–no-mixed” and “–no-discordant” parameters for NPC dataset)^66^. The aligned reads were summarized using the “featureCounts” function of the RSubread (v.2.6.4) R/Bioconductor package^66^ and the reads were assigned using the hg38 GTF annotation from the UCSC database^67^(parameters: isPairedEnd=isPairedEnd=autosort = T, allowMultiOverlap = F, strandSpecific=2 for NPC dataset and ignoreDup=FALSE, useMetaFeatures=TRUE, countMultiMappingReads=FALSE for post-mortem brain dataset). A summary of the read counts at each preprocessing step was plotted using ggplot2 (v.3.3.6) (Figs. S1a and S2a). Expression of TSC1 was further assessed in the NPC data set using TMM-log2 normalized counts obtained by running calcNormFactors function from edgeR (v.3.34.1)^48^ and voom function from limma (v.3.48.3)^66^ (Fig. S1b). Principal component analysis (PCA) on previously obtained normalized counts^48^ was performed using the PCAtools R package (v. 2.4.0) https://github.com/kevinblighe/PCAtools.) (parameters removeVar = 0.75 and scale = T) and visualized using eigencorplot,screeplot and biplot functions from the PCAtools R package (v.2.4.0) https://ggplot2.tidyverse.org.) (Figs. S1c-e and S2b-d).

### Gene ontology analysis

Genes identified as regulated via the “translation” mode in anota2seq (i.e. transcripts with an increase or decrease in polysome-associated mRNA levels without corresponding changes in total cytosolic mRNA levels) were used as input for gene-set enrichment analysis using Cytoscape (v.3.8.2.) plug-in ClueGO (v.2.5.8)^47^ with p-value cutoff = 0.001 or 0.01 for NPCs and post-mortem brain samples, respectively, together with p-value cutoff = True, Correction Method Used = Benjamini-Hochberg, Statistical Test Used = Enrichment (Right-sided hypergeometric test), Kappa= 0.4, Min. Percentage = 10, Min GO Level = 7, Max GO Level = 15, Number of Genes = 3, GO Fusion = false, GO Group = true, Over View Term = SmallestPValue, Group By Kapp Statistics = true, Initial Group Size = 1, Sharing Group Percentage = 50.0, Ontology Used = GO_BiologicalProcess-EBI-UniProt-GOA-ACAP ARAP_13.05.2021_00h00, KEGG_13.05.2021, REACTOME_Reactions_13.05.2021, Evidence codes used = All, Identifiers used = SymbolID. The resulting networks were set to show the most significant term identified for each group.

### Analysis of gene signatures using empirical cumulative distribution functions

To cross-compare RNA-seq datasets, empirical cumulative distribution functions (ECDFs) of log2 fold changes for polysome-associated and cytosolic mRNA were plotted independently for genes that were found to be translationally regulated in either data set. The difference between each tested gene set and the background was quantified at the 50th quantile and the Wilcoxon rank-sum test was used to determine whether there was a significant shift between the background and each signature. The same approach was used to assess signatures of transcripts whose translation was previously identified as increased upon eIF4E over-expression^50^ or genes associated with synaptic function^49^.

### Nanostring nCounter Gene Expression Analysis

#### Target gene selection and generation of custom nanostring panel

For NanoString nCounter analysis^68^, a custom panel of 200 target genes identified by Anota2seq analysis as regulated by “translation” or “mRNA abundance” were selected: (i) genes with log_2_FC > 2 and FDR < 0.15 in any of the contrasts applied when analyzing the NPC data set, (ii) targets annotated to ASD/NDD pathology with log2FC > 1 and FDR < 0.15 in the NPC data set and (iii) negative controls were identified based on standard deviation (<0.3) between samples, mean log2 TMM signal in the top 50th quartile, deltaT <0.1 (from anota2seq analysis) and deltaP <0.1 (from anota2seq analysis).

#### Sample preparation and data analysis

RNA integrity for polysome-associated and cytosolic mRNA (4 replicates of each condition) was validated using the RNA NanoChip on an Agilent 2100 Bioanalyzer (Agilent Technology, Santa Clara, CA). Concentration of RNA was measured using the QubitTM RNA HS Assay Kit (Invitrogen, Carlsbad, CA). Samples from each condition were randomized on cartridges and processed by the KIGene Core Facility (Karolinska Institute, Sweden) using 100 ng input for cytosolic mRNA and 300 ng input for polysome-associated mRNA. The “newRccSet” function from the NanoStringQCPro (v.1.24.0) R/Bioconductor package was used to pre-process raw data. Genes with expression less than 6.27 (log2 scale; Fig. 1d data set) and 5.63 (Fig. 5c–d data set) in 3 or more samples were excluded (thresholds were determined by calculating the mean log2 expression level of negative control genes + 2 standard deviation). This resulted in analysis of 175 and 164 transcripts for Fig. 1d and Fig. 5c–d data sets, respectively. For Fig. 1d analysis, the geNorm function of the CtrlGene (v.1.0.1) package was used to identify housekeeping genes for normalization. *BUD31, BASP1*, and *GAB2* were chosen for the *TSC1*^*−/−*^ vs *TSC1*^+/+^ comparison. The data was normalized using the contentNorm function (from the NanoStringQCPro package) with the following parameters: method = “housekeeping”, summaryFunction=“mean” and hk= a vector containing housekeeping gene names. Following the housekeeping normalization, an additional step of variance stabilizing normalization (vsn) was performed using justvsn function from the vsn(v.3.60.0)^69^ R/Bioconductor package. For the Fig. 5c–d data set comparing mTORC1 inhibitors, no housekeeping genes could be identified (possibly as the RMC-6272 treatment was not included during selection of housekeeping genes) Therefore, global normalization was performed using contentNorm function with the following parameters: method = “housekeeping”, summaryFunction=”mean”. Similar to above, vsn normalization was then performed. Log2 fold changes were then calculated and plotted (Figs. 1d and 5c–d).

### Validation of differential translation using RT-qPCR

To validate using RT-qPCR, polysomes from *TSC1*^−/−^ or *TSC1*^+/+^ NPCs (3 biological replicates) were fractionated and pooled as described for NPCs above. RNA was extracted using TRIzol and cDNA prepared using M-MuLV Reverse Transcriptase (New England Biolabs, Ipswich, MA) and oligo(dT)20 primers. qPCRs were performed with SsoFast Evagreen Supermix (Bio-Rad, Hercules, CA) using the CFX96 PCR system (Bio-Rad Hercules, CA). Primers for RT-qPCR are detailed in Table S4. The level of each mRNA was normalized to β-actin (*ACTB*) using the comparative CT method and compared across conditions as indicated in figure legends.

### Cell size, proliferation and neurite outgrowth assays

For cell size, proliferation, and neurite outgrowth, assays were performed as previously described^17^ . Briefly, cell size and proliferation were determined from live cells (3 biological replicates/treatment condition) by trypan blue exclusion using the Countess II automated cell counter (ThermoFisher, Waltham, MA). For neurite outgrowth, NPCs (6250 per cm^2^) were seeded on Poly-D-lysine coated wells (0.1 mg/ml; Sigma, St. Louis, MO) and Fibronectin (5 μg/ml, Corning, Corning, NY) in growth factor-depleted neural expansion medium (30% NEM) containing 1:1 of neurobasal media and advanced DMEM/F12 (ThermoFisher, Waltham, MA), 1X penicillin/streptomycin and 0.3X neural induction supplement (ThermoFisher, Waltham, MA). Cells were grown in presence of DMSO, 50 nM rapamycin, or 10 nM of RMC-6272 for 48 h and fixed with 4% paraformaldehyde (PFA; Microscopy Sciences, Hatfield, PA) for 20 min prior to immunostaining. Cover slips from 3 biological replicates were analyzed. For each cell line, images from 4 independent fields/condition (~50 cells/field) were acquired for analysis. Processes that were at least two times the length of the cell body were considered as neurites. The average neurite number per cell and the average neurite length per cell were analyzed using HCA-Vision software V.2.2.0 (CSIRO, Canberra, Australia).

### Immunocytochemistry

Cells were fixed with 4% PFA for 20 min at room temperature and washed 3 times with PBS. Non-specific labeling was blocked, and cell membranes permeabilized in a single step, using 4% normal goat serum (NGS) in PBS containing 0.1% Triton-X-100 and 0.05%Tween-20 for 45 min at room temperature. Primary antibodies were diluted in 2% NGS/0.1%Triton-X-100/PBS and incubated for 2 h in the dark at room temperature (see Table S2 for primary antibodies). Coverslips were mounted in ProLong Gold antifade reagent with DAPI (Invitrogen, Carlsbad, CA) and immunofluorescence was visualized on a Nikon Eclipse TE2000-U microscope. Images were acquired using a Nikon DS-QiMc camera and NIS-Element BR 3.2 imaging software.

### Immunoblot analyses

Immunoblotting was performed as previously described^17^. Briefly, cells were lysed in RIPA buffer, and protein lysates were resolved on Novex 4–12% or 10–20% Tris-Glycine gels (Invitrogen, Carlsbad, CA), transferred to nitrocellulose (Bio-Rad, Hercules, CA) and then incubated with primary antibodies (see Table S2 for primary antibodies). All immunoblotting data shown is a representative of at least 3 biological replicates.

### Statistical information

For RNA sequencing, statistical analyses from 3 biological replicates were performed using RStudio (R v.4.1.1). Changes in translational efficiency were assessed using batch-adjusted analysis of partial variance (APV) in combination with a random variance model implemented in the anota2seq bioconductor package. The p-values obtained from the analysis were adjusted using the Benjamini-Hochberg (BH) method. ECDFs were used to cross-compare RNA-seq datasets and assess selective regulation of signatures, and significance was assessed using the Wilcoxon rank-sum test relative to the background. Right-sided hypergeometric tests were used to identify GO terms enriched for genes identified by anota2seq with a p-value cutoff 0.001 or 0.01 for NPCs and post-mortem brain samples respectively; and the obtained p-values were adjusted using the BH method. All tests were two-tailed unless otherwise indicated. For cell size and proliferation, p-values were determined from 3 biological replicates by one-tailed Student’s t-test. For neurite outgrowth assays, quantitation from 3 biological replicates was performed from using HCA-Vision software, and p-values were calculated by one-tailed Student’s t test.

## References

[1] Caban C., Khan N., Hasbani D. M. & Crino P. B. Genetics of tuberous sclerosis complex: implications for clinical practice. Appl Clin Genet 10, 1–8, doi:10.2147/TACG.S90262 (2017). 28053551 PMC5189696

[2] Henske E. P., Jozwiak S., Kingswood J. C., Sampson J. R. & Thiele E. A. Tuberous sclerosis complex. Nat Rev Dis Primers 2, 16035, doi:10.1038/nrdp.2016.35 (2016). 27226234

[3] Han J. M. & Sahin M. TSC1/TSC2 signaling in the CNS. FEBS letters 585, 973–980, doi:10.1016/j.febslet.2011.02.001 (2011). 21329690 PMC3070766

[4] Huang J. & Manning B. D. The TSC1-TSC2 complex: a molecular switchboard controlling cell growth. Biochem J 412, 179–190 (2008). 18466115 10.1042/BJ20080281 PMC2735030

[5] Bissler J. J. Sirolimus for angiomyolipoma in tuberous sclerosis complex or lymphangioleiomyomatosis. N Engl J Med 358, 140–151 (2008). 18184959 10.1056/NEJMoa063564 PMC3398441

[6] Davies D. M. Sirolimus therapy for angiomyolipoma in tuberous sclerosis and sporadic lymphangioleiomyomatosis: a phase 2 trial. Clinical cancer research : an official journal of the American Association for Cancer Research 17, 4071–4081, doi:10.1158/1078-0432.CCR-11-0445 (2011). 21525172

[7] Krueger D. A. Everolimus for subependymal giant-cell astrocytomas in tuberous sclerosis. The New England journal of medicine 363, 1801–1811, doi:10.1056/NEJMoa1001671 (2010). 21047224

[8] Julich K. & Sahin M. Mechanism-based treatment in tuberous sclerosis complex. Pediatric neurology 50, 290–296, doi:10.1016/j.pediatrneurol.2013.12.002 (2014). 24486221 PMC3959246

[9] Overwater I. E., Rietman A. B., van Eeghen A. M. & de Wit M. C. Y. Everolimus for the treatment of refractory seizures associated with tuberous sclerosis complex (TSC): current perspectives. Ther Clin Risk Manag 15, 951–955, doi:10.2147/TCRM.S145630 (2019). 31440057 PMC6666377

[10] Overwater I. E. A randomized controlled trial with everolimus for IQ and autism in tuberous sclerosis complex. Neurology 93, e200–e209, doi:10.1212/WNL.0000000000007749 (2019). 31217257

[11] Krueger D. A. Everolimus for treatment of tuberous sclerosis complex-associated neuropsychiatric disorders. Ann Clin Transl Neurol 4, 877–887, doi:10.1002/acn3.494 (2017). 29296616 PMC5740257

[12] Sahin M. Advances and Future Directions for Tuberous Sclerosis Complex Research: Recommendations From the 2015 Strategic Planning Conference. Pediatr Neurol 60, 1–12, doi:10.1016/j.pediatrneurol.2016.03.015 (2016). 27267556 PMC4921275

[13] Zucco A. J. Neural progenitors derived from Tuberous Sclerosis Complex patients exhibit attenuated PI3K/AKT signaling and delayed neuronal differentiation. Mol Cell Neurosci 92, 149–163, doi:10.1016/j.mcn.2018.08.004 (2018). 30144504 PMC6250058

[14] Winden K. D. Biallelic Mutations in TSC2 Lead to Abnormalities Associated with Cortical Tubers in Human iPSC-Derived Neurons. J Neurosci 39, 9294–9305, doi:10.1523/JNEUROSCI.0642-19.2019 (2019). 31591157 PMC6867816

[15] Sundberg M. Purkinje cells derived from TSC patients display hypoexcitability and synaptic deficits associated with reduced FMRP levels and reversed by rapamycin. Mol Psychiatry 23, 2167–2183, doi:10.1038/s41380-018-0018-4 (2018). 29449635 PMC6093816

[16] Nadadhur A. G. Neuron-Glia Interactions Increase Neuronal Phenotypes in Tuberous Sclerosis Complex Patient iPSC-Derived Models. Stem Cell Reports 12, 42–56, doi:10.1016/j.stemcr.2018.11.019 (2019). 30581017 PMC6335594

[17] Martin P. TSC patient-derived isogenic neural progenitor cells reveal altered early neurodevelopmental phenotypes and rapamycin-induced MNK-eIF4E signaling. Mol Autism 11, 2, doi:10.1186/s13229-019-0311-3 (2020). 31921404 PMC6945400

[18] Li Y. Abnormal Neural Progenitor Cells Differentiated from Induced Pluripotent Stem Cells Partially Mimicked Development of TSC2 Neurological Abnormalities. Stem Cell Reports 8, 883–893, doi:10.1016/j.stemcr.2017.02.020 (2017). 28344003 PMC5390135

[19] Grabole N. Genomic analysis of the molecular neuropathology of tuberous sclerosis using a human stem cell model. Genome Med 8, 94, doi:10.1186/s13073-016-0347-3 (2016). 27655340 PMC5031259

[20] Costa V. mTORC1 Inhibition Corrects Neurodevelopmental and Synaptic Alterations in a Human Stem Cell Model of Tuberous Sclerosis. Cell Rep 15, 86–95, doi:10.1016/j.celrep.2016.02.090 (2016). 27052171

[21] Blair J. D., Hockemeyer D. & Bateup H. S. Genetically engineered human cortical spheroid models of tuberous sclerosis. Nat Med 24, 1568–1578, doi:10.1038/s41591-018-0139-y (2018). 30127391 PMC6261470

[22] Blair J. D. & Bateup H. S. New frontiers in modeling tuberous sclerosis with human stem cell-derived neurons and brain organoids. Dev Dyn 249, 46–55, doi:10.1002/dvdy.60 (2020). 31070828 PMC6995669

[23] Afshar Saber W. & Sahin M. Recent advances in human stem cell-based modeling of Tuberous Sclerosis Complex. Mol Autism 11, 16, doi:10.1186/s13229-020-0320-2 (2020). 32075691 PMC7031912

[24] Liu G. Y. & Sabatini D. M. mTOR at the nexus of nutrition, growth, ageing and disease. Nat Rev Mol Cell Biol 21, 183–203, doi:10.1038/s41580-019-0199-y (2020). 31937935 PMC7102936

[25] Halbeisen R. E., Scherrer T. & Gerber A. P. Affinity purification of ribosomes to access the translatome. Methods 48, 306–310, doi:10.1016/j.ymeth.2009.04.003 (2009). 19398006

[26] Wang Z. Y. Transcriptome and translatome co-evolution in mammals. Nature 588, 642–647, doi:10.1038/s41586-020-2899-z (2020). 33177713 PMC7116861

[27] Tebaldi T. Widespread uncoupling between transcriptome and translatome variations after a stimulus in mammalian cells. BMC Genomics 13, 220, doi:10.1186/1471-2164-13-220 (2012). 22672192 PMC3441405

[28] Larsson O., Tian B. & Sonenberg N. Toward a genome-wide landscape of translational control. Cold Spring Harb Perspect Biol 5, a012302, doi:10.1101/cshperspect.a012302 (2013). 23209130 PMC3579401

[29] Gingras A. C., Raught B. & Sonenberg N. eIF4 initiation factors: effectors of mRNA recruitment to ribosomes and regulators of translation. Annu Rev Biochem 68, 913–963, doi:10.1146/annurev.biochem.68.1.913 (1999). 10872469

[30] Holz M. K., Ballif B. A., Gygi S. P. & Blenis J. mTOR and S6K1 mediate assembly of the translation preinitiation complex through dynamic protein interchange and ordered phosphorylation events. Cell 123, 569–580, doi:10.1016/j.cell.2005.10.024 (2005). 16286006

[31] Raught B. Phosphorylation of eucaryotic translation initiation factor 4B Ser422 is modulated by S6 kinases. EMBO J 23, 1761–1769, doi:10.1038/sj.emboj.7600193 (2004). 15071500 PMC394247

[32] Blair J. D., Hockemeyer D., Doudna J. A., Bateup H. S. & Floor S. N. Widespread Translational Remodeling during Human Neuronal Differentiation. Cell Rep 21, 2005–2016, doi:10.1016/j.celrep.2017.10.095 (2017). 29141229 PMC5759054

[33] Roux P. P. & Topisirovic I. Signaling Pathways Involved in the Regulation of mRNA Translation. Mol Cell Biol 38, doi:10.1128/MCB.00070-18 (2018). PMC5974435 29610153

[34] Brennand K. Phenotypic differences in hiPSC NPCs derived from patients with schizophrenia. Mol Psychiatry 20, 361–368, doi:10.1038/mp.2014.22 (2015). 24686136 PMC4182344

[35] Kaushik G. & Zarbalis K. S. Prenatal Neurogenesis in Autism Spectrum Disorders. Front Chem 4, 12, doi:10.3389/fchem.2016.00012 (2016). 27014681 PMC4791366

[36] Marchetto M. C. Altered proliferation and networks in neural cells derived from idiopathic autistic individuals. Mol Psychiatry 22, 820–835, doi:10.1038/mp.2016.95 (2017). 27378147 PMC5215991

[37] Packer A. Neocortical neurogenesis and the etiology of autism spectrum disorder. Neurosci Biobehav Rev 64, 185–195, doi:10.1016/j.neubiorev.2016.03.002 (2016). 26949225

[38] Mellios N. MeCP2-regulated miRNAs control early human neurogenesis through differential effects on ERK and AKT signaling. Mol Psychiatry 23, 1051–1065, doi:10.1038/mp.2017.86 (2018). 28439102 PMC5815944

[39] Sheridan S. D. Epigenetic characterization of the FMR1 gene and aberrant neurodevelopment in human induced pluripotent stem cell models of fragile X syndrome. PloS one 6, e26203, doi:10.1371/journal.pone.0026203 (2011). 22022567 PMC3192166

[40] Williams M. Rapid Detection of Neurodevelopmental Phenotypes in Human Neural Precursor Cells (NPCs). J Vis Exp, doi:10.3791/56628 (2018). PMC5931427 29553565

[41] Burnett G. L. Discovery of RMC-5552, a Selective Bi-Steric Inhibitor of mTORC1, for the Treatment of mTORC1-Activated Tumors. J Med Chem 66, 149–169, doi:10.1021/acs.jmedchem.2c01658 (2023). 36533617 PMC9841523

[42] Ristau J., Watt K., Oertlin C. & Larsson O. Polysome Fractionation for Transcriptome-Wide Studies of mRNA Translation. Methods Mol Biol 2418, 223–241, doi:10.1007/978-1-0716-1920-9_14 (2022). 35119669

[43] Oertlin C., Watt K., Ristau J. & Larsson O. Anota2seq Analysis for Transcriptome-Wide Studies of mRNA Translation. Methods Mol Biol 2418, 243–268, doi:10.1007/978-1-0716-1920-9_15 (2022). 35119670

[44] Oertlin C. Generally applicable transcriptome-wide analysis of translation using anota2seq. Nucleic Acids Res 47, e70, doi:10.1093/nar/gkz223 (2019). 30926999 PMC6614820

[45] Lorent J. Translational offsetting as a mode of estrogen receptor alpha-dependent regulation of gene expression. EMBO J 38, e101323, doi:10.15252/embj.2018101323 (2019). 31556460 PMC6885737

[46] Kusnadi E. P., Timpone C., Topisirovic I., Larsson O. & Furic L. Regulation of gene expression via translational buffering. Biochim Biophys Acta Mol Cell Res 1869, 119140, doi:10.1016/j.bbamcr.2021.119140 (2022). 34599983

[47] Bindea G. ClueGO: a Cytoscape plug-in to decipher functionally grouped gene ontology and pathway annotation networks. Bioinformatics 25, 1091–1093, doi:10.1093/bioinformatics/btp101 (2009). 19237447 PMC2666812

[48] Liang S. Polysome-profiling in small tissue samples. Nucleic Acids Res 46, e3, doi:10.1093/nar/gkx940 (2018). 29069469 PMC5758873

[49] Gupta S. Transcriptome analysis reveals dysregulation of innate immune response genes and neuronal activity-dependent genes in autism. Nat Commun 5, 5748, doi:10.1038/ncomms6748 (2014). 25494366 PMC4270294

[50] Larsson O. Eukaryotic translation initiation factor 4E induced progression of primary human mammary epithelial cells along the cancer pathway is associated with targeted translational deregulation of oncogenic drivers and inhibitors. Cancer Res 67, 6814–6824, doi:10.1158/0008-5472.CAN-07-0752 (2007). 17638893

[51] Masvidal L., Hulea L., Furic L., Topisirovic I. & Larsson O. mTOR-sensitive translation: Cleared fog reveals more trees. RNA Biol 14, 1299–1305, doi:10.1080/15476286.2017.1290041 (2017). 28277937 PMC5711451

[52] Larsson O. Distinct perturbation of the translatome by the antidiabetic drug metformin. Proc Natl Acad Sci U S A 109, 8977–8982, doi:10.1073/pnas.1201689109 (2012). 22611195 PMC3384216

[53] Lee B. J. Selective inhibitors of mTORC1 activate 4EBP1 and suppress tumor growth. Nat Chem Biol 17, 1065–1074, doi:10.1038/s41589-021-00813-7 (2021). 34168367 PMC9249104

[54] Lee B. J. Efficacy of a Novel Bi-Steric mTORC1 Inhibitor in Models of B-Cell Acute Lymphoblastic Leukemia. Front Oncol 11, 673213, doi:10.3389/fonc.2021.673213 (2021). 34408976 PMC8366290

[55] Tahmasebi S., Khoutorsky A., Mathews M. B. & Sonenberg N. Translation deregulation in human disease. Nat Rev Mol Cell Biol 19, 791–807, doi:10.1038/s41580-018-0034-x (2018). 30038383

[56] Saba J. A., Liakath-Ali K., Green R. & Watt F. M. Translational control of stem cell function. Nat Rev Mol Cell Biol 22, 671–690, doi:10.1038/s41580-021-00386-2 (2021). 34272502

[57] Bilanges B. Tuberous sclerosis complex proteins 1 and 2 control serum-dependent translation in a TOP-dependent and -independent manner. Mol Cell Biol 27, 5746–5764, doi:10.1128/MCB.02136-06 (2007). 17562867 PMC1952130

[58] Fan Q. W., Nicolaides T. P. & Weiss W. A. Inhibiting 4EBP1 in Glioblastoma. Clin Cancer Res 24, 14–21, doi:10.1158/1078-0432.CCR-17-0042 (2018). 28696243 PMC5754225

[59] Winden K. D., Ebrahimi-Fakhari D. & Sahin M. Abnormal mTOR Activation in Autism. Annu Rev Neurosci 41, 1–23, doi:10.1146/annurev-neuro-080317-061747 (2018). 29490194

[60] Pagani M. mTOR-related synaptic pathology causes autism spectrum disorder-associated functional hyperconnectivity. Nat Commun 12, 6084, doi:10.1038/s41467-021-26131-z (2021). 34667149 PMC8526836

[61] Ingolia N. T., Hussmann J. A. & Weissman J. S. Ribosome Profiling: Global Views of Translation. Cold Spring Harb Perspect Biol 11, doi:10.1101/cshperspect.a032698 (2019). PMC6496350 30037969

[62] Fu J. M. Rare coding variation provides insight into the genetic architecture and phenotypic context of autism. Nat Genet 54, 1320–1331, doi:10.1038/s41588-022-01104-0 (2022). 35982160 PMC9653013

[63] Amorim I. S., Lach G. & Gkogkas C. G. The Role of the Eukaryotic Translation Initiation Factor 4E (eIF4E) in Neuropsychiatric Disorders. Front Genet 9, 561, doi:10.3389/fgene.2018.00561 (2018). 30532767 PMC6265315

[64] Steinberger J. Identification and characterization of hippuristanol-resistant mutants reveals eIF4A1 dependencies within mRNA 5’ leader regions. Nucleic Acids Res 48, 9521–9537, doi:10.1093/nar/gkaa662 (2020). 32766783 PMC7515738

[65] Quast C. The SILVA ribosomal RNA gene database project: improved data processing and web-based tools. Nucleic Acids Res 41, D590–596, doi:10.1093/nar/gks1219 (2013). 23193283 PMC3531112

[66] Liao Y., Smyth G. K. & Shi W. The R package Rsubread is easier, faster, cheaper and better for alignment and quantification of RNA sequencing reads. Nucleic Acids Res 47, e47, doi:10.1093/nar/gkz114 (2019). 30783653 PMC6486549

[67] Karolchik D. The UCSC Table Browser data retrieval tool. Nucleic Acids Res 32, D493–496, doi:10.1093/nar/gkh103 (2004). 14681465 PMC308837

[68] Geiss G. K. Direct multiplexed measurement of gene expression with color-coded probe pairs. Nat Biotechnol 26, 317–325, doi:10.1038/nbt1385 (2008). 18278033

[69] Huber W., von Heydebreck A., Sultmann H., Poustka A. & Vingron M. Variance stabilization applied to microarray data calibration and to the quantification of differential expression. Bioinformatics 18 Suppl 1, S96–104, doi:10.1093/bioinformatics/18.suppl_1.s96 (2002). 12169536

